# ﻿Evidence for further non-coding RNA genes in the fungal rDNA region

**DOI:** 10.3897/mycokeys.90.84866

**Published:** 2022-06-30

**Authors:** Magnus Alm Rosenblad, Ellen Larsson, Arttapon Walker, Naritsada Thongklang, Christian Wurzbacher, R. Henrik Nilsson

**Affiliations:** 1 Department of Chemistry and Molecular Biology, National Infrastructure of Bioinformatics (NBIS), Lundberg laboratory, University of Gothenburg, Gothenburg, Sweden; 2 Gothenburg Global Biodiversity Centre, Department of Biological and Environmental Sciences, University of Gothenburg, Box 461, 405 30 Gothenburg, Sweden; 3 Center of Excellence in Fungal Research, Mae Fah Luang University, Chiang Rai 57100, Thailand; 4 School of Science, Mae Fah Luang University, Chiang Rai 57100, Thailand; 5 Chair of Urban Water Systems Engineering, Technical University of Munich, Am Coulombwall 3, 85748 Garching, Germany

**Keywords:** Basidiomycetes, IGS, ITS, MRP, non-coding RNA, RNase MRP, RNase P, SRP

## Abstract

Non-coding RNA (ncRNA) genes play important, but incompletely understood, roles in various cellular processes, notably translation and gene regulation. A recent report on the detection of the ncRNA Signal Recognition Particle gene in the nuclear ribosomal internal transcribed spacer region of several species of three genera of ectomycorrhizal basidiomycetes prompted a more thorough bioinformatics search for additional ncRNA genes in the full fungal ribosomal operon. This study reports on the detection of three ncRNA genes hitherto not known from the fungal ribosomal region: nuclear RNase P RNA, RNase MRP RNA, and a possible snoRNA U14 in a total of five species of *Auricularia* and *Inocybe*. We verified their presence through resequencing of independent specimens. Two completed *Auricularia* genomes were found to lack these ncRNAs elsewhere than in the ribosomal operon, suggesting that these are functional genes. It seems clear that ncRNA genes play a larger role in fungal ribosomal genetics than hitherto thought.

## ﻿Introduction

Non-coding RNA (ncRNA) are stretches of RNA – typically thought of as genes – that are not translated into proteins through translation. A range of functions has been ascribed to the various groups of ncRNAs known to date, including important roles in translation, gene regulation, and chromosome inactivation (Cech and Steitz 2014). The number of ncRNA genes in the human genome alone is believed to run in the thousands, although relatively few have been characterised to any satisfactory level (Li and Liu 2019). Knowledge of ncRNAs in fungal genomes is rudimentary by comparison, but ncRNAs appear to play important cellular roles in the relatively few fungi examined to date (Li et al. 2021). The ncRNAs identified in the present study are, with few exceptions, all ubiquitous in eukaryotes and there are as yet no examples of them missing in fungi. They play important roles in tRNA processing (RNase P RNA), rRNA maturation (RNase MRP RNA), and ER targeting of proteins (SRP RNA), and they constitute the RNA component of the respective ribonucleoprotein (RNP) particles (López et al. 2009; Akopian et al. 2013).

Alm Rosenblad et al. (2016) unexpectedly found sequence analysis-derived evidence of the ncRNA Signal Recognition Particle (SRP) RNAs in the nuclear ribosomal ITS1 region of 11 species in three ectomycorrhizal genera of the Basidiomycota: *Astraeus*, *Russula*, and *Lactarius*. Indirect evidence furthermore suggested that these SRP RNAs could be functional. Queries in the GenBank (Sayers et al. 2021) and UNITE (Nilsson et al. 2019; Abarenkov et al. 2022) databases failed to produce any other fungi with SRP RNA in their ITS region, raising questions as to why such a rare genetic element would have been gained several times independently in the ITS1 region of a set of three relatively closely related genera.

The recent trend of employing various high-throughput sequencing technologies to generate longer stretches of the fungal ribosomal operon than just the ITS region (Wurzbacher et al. 2019) offers a possibility to extend the search for fungal ncRNAs beyond the ITS region. The present study reports on a broadening of the search of Alm Rosenblad et al. (2016) to include a wider selection of ncRNAs and to cover the full nuclear ribosomal operon. We recovered and verified three to four different ncRNAs from the ribosomal intergenic spacer 1 (IGS1) region of a set of *Auricularia* and *Inocybe* species, and we submit that ncRNAs are elements that can no longer be disregarded in the context of fungal ribosomal biology.

## ﻿Materials and methods

### ﻿Sequence query

Since ncRNAs are conserved primarily on the secondary structure level rather than the primary sequence level, we queried GenBank for fungal ribosomal ncRNAs using the secondary structure covariance models from the Rfam database (Nawrocki et al. 2014) and Dumesic et al. (2015) through the cmsearch and cmscan commands of the INFERNAL v1.1 package (Nawrocki and Eddy 2013). As a part of an ongoing phylogenetic study of the basidiomycete genus *Inocybe*, we also sequenced the full ribosomal operon of four *Inocybe* species: *Inocybecincinnata*, *Inocybeflocculosa*, *Inocybeleiocephala*, and *Inocybephaeocystidiosa*. We queried these, too, for any new ncRNAs. We considered only highly significant matches that passed the Rfam E-value threshold applied for each gene. We then double-checked all matches by detailed manual examination of all conserved motifs as well as the secondary structure to filter out any partial or spurious candidates.

### ﻿Verification of ncRNA matches

After quality control filtering, our GenBank query produced more than 30 highly significant ncRNA matches belonging to four different ncRNA genes in the IGS1 region of several *Auricularia* species from Li et al. (2011). Attempts at locating the underlying fungal specimens through the publication were unsuccessful. However, other Asian specimens from the same and related species were located in the Mae Fah Luang University herbarium (Table 1). To rule out sequencing or assembly error by the initial sequence authors, we thus ordered and sequenced those specimens for the full ribosomal operon.

We targeted two species of *Auricularia* and four species of *Inocybe* for sequencing of the full ribosomal operon. The fungal DNA was extracted using a DNA plant Mini Kit (Qiagen) and subsequently amplified using the primers NS1rc and RCA95rc or Fun-rOP-F/Fun-rOP-R as detailed in Wurzbacher et al. (2019). Briefly, the long-range PCR was performed using the PrimeStar GLX polymerase (Takara) with a 2.5 min elongation time for the first primer pair, and a 4 min elongation time for the second primer pair for 36 cycles. The samples were subsequently barcoded by an index PCR with 10 additional cycles. The amplicons were then sequenced with either a MinION instrument (Oxford Nanopore Technologies; LSK-308 library preparation; R9.5 flow cell) or sequenced in circular consensus mode with a PacBio RSII (Pacific Biosciences). The generated sequence data were processed as outlined in Wurzbacher et al. (2019; Suppl. material 1) using a quality filtering step, demultiplexing, alignment, clustering, and consensus generation. The newly generated *Auricularia* and *Inocybe* consensus sequences were queried for the presence of ncRNAs as detailed above.

### ﻿Assessment of ncRNA functionality in *Auricularia*

An opportunity to at least partially assess whether the ncRNAs found in the *Auricularia* specimens may be functional – rather than pseudogenes – presented itself through the draft genome assemblies of *Auriculariaheimuer* (strain Dai 13782; NCBI WGS accession NEKD01; Fang et al. 2020) and *Auriculariacornea* (strain CCMJ2827, WGS RJDY01; Dai et al. 2019). If these genomes, too, were found to contain these ncRNAs in the ribosomal operon, but nowhere else in the genome, then that would suggest that those ncRNA copies are functional. The draft assemblies were queried with the Rfam covariance models as above.

## ﻿Results

### ﻿Sequence query and verification of ncRNA matches

**Figure 1.**
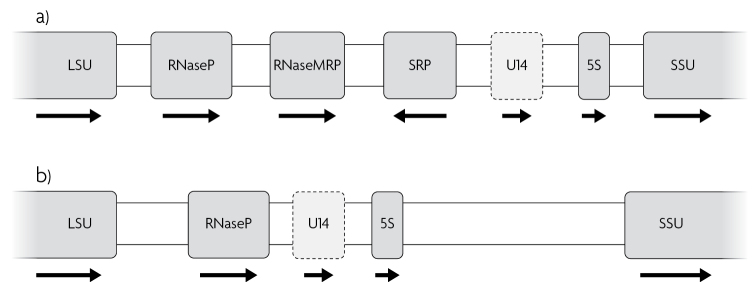
Schematic illustration of the fungal IGS region and neighbouring genes. Shown are **a***Auriculariacornea* and **b***Inocybeleiocephala*. The four ncRNA elements SRP RNA, nuclear RNase P RNA, RNase MRP RNA, and U14 are shown. U14 is shown in dashed outline to indicate its somewhat hypothetical nature. The arrows indicate strand. This schematic figure is not fully drawn to scale. The distance between LSU and SSU is approximately 5,000 bases, the length of U14 is approximately 200 bases, and the length of the other ncRNAs is approximately 300 bases.

Our GenBank query produced more than 30 highly significant ncRNA matches (belonging to the four different ncRNA genes SRP RNA, nuclear RNase P RNA, RNase MRP RNA, and a possible U14) in the IGS1 region of several *Auricularia* species in GenBank (Fig. 1; Table 1; Suppl. material 2). We re-sequenced the full ribosomal operon of two of these *Auricularia* species plus four *Inocybe* species. An ncRNA query of the newly generated sequences verified that the same ncRNAs were present in the same order in these independent specimens, showing that the original GenBank sequences did not represent mis-assemblies or otherwise artifactual sequence data. Interestingly, another *Auricularia* species – *Auriculariapolytricha* strain MG66 – was found to lack these ncRNAs altogether in the ribosomal operon. Similarly, we found a different combination of ncRNAs (nuclear RNase P and U14) in the IGS1 of *Inocybecincinnata*, *Inocybeflocculosa*, and *Inocybeleiocephala*; however, *Inocybephaeocystidiosa* was found to lack all of the above ncRNAs in the ribosomal operon (Fig. 1, Table 1).

**Table 1.** List of specimens/sequences with at least one ncRNA beyond the ordinary rRNA genes. GenBank and collection/herbarium accession numbers are shown. The columns SRP, nuclear RNase P, RNase MRP, and U14 indicate whether these genes were recovered in the ribosomal operon of the specimen/sequence in question. The majority of the *Auriculariaauricula-judae* sequences are from Li et al. (2014). a) For entry WGS:NEKD01, the contig NEKD01000094 contains four operons covering SSU-5S. b) For entry WGS:AFVO01, no rRNA operon could be found in the assembly. c) For entry WGS:QFEN01, the ncRNA genes were found on separate contigs (viz. nuclear RNase P in QFEN01000681, RNase MRP in QFEN01000187, and SRP in QFEN01000909). d) For entry WGS:RJDY01, the ncRNA genes are found in the three operons in RJDY01000048. Accession numbers given in bold were produced as part of this study. WGS project identifiers refer to the NCBI Whole Genome Shotgun assembly database.

GenBank	Species name	Voucher specimen	nuclear RNase P	RNase MRP	SRP	U14
**OM964555**	*Inocybecincinnata*	EL113-16	Y	N	N	Y
**OM964556**	*Inocybeflocculosa*	EL168-16	Y	N	N	Y
**OM964554**	*Inocybeleiocephala*	EL85-16	Y	N	N	Y
**OM964557**	*Inocybephaeocystidiosa*	EL23-16	N	N	N	N
**OM964558**	*Auriculariacornea*	MFLU16-2108	Y	Y	Y	Y
**OM964559**	*Auriculariadelicata*	MFLU16-2118	Y	Y	Y	Y
WGS:NEKD01	*Auriculariaheimuer* (a)	Dai 13782	Y	Y	Y	Y
WGS:AFVO01	*Auriculariasubglabra* (b)	TFB-10046 SS5	N	N	N	N
WGS:QFEN01	*Auriculariapolytricha* (c)	MG66	Y	Y	Y	N
JF440699.1	*Auriculariapolytricha*	AP112	Y	Y	Y	Y
JF440698.1	*Auriculariapolytricha*	APFJ	Y	Y	Y	Y
JF440701.1	*Auriculariadelicata*	ADFJ	Y	Y	Y	Y
JF440702.1	*Auriculariadelicata*	AD5424	Y	Y	Y	Y
JF440697.1	*Auriculariafuscosuccinea*	AFJLH	Y	Y	Y	Y
JF440700.1	*Auriculariapeltata*	APLME	Y	Y	Y	Y
MN156315	*Auriculariacornea*	B02	Y	Y	Y	Y
WGS:RJDY01	*Auriculariacornea* (d)	CCMJ2827	Y	Y	Y	Y
HQ414239.1	*Auriculariaauricula-judae*	XK-1	Y	Y	Y	Y
HQ414240.1	*Auriculariaauricula-judae*	HE-1	Y	Y	Y	Y
HQ414241.1	*Auriculariaauricula-judae*	DP-5	Y	Y	Y	Y
HQ414242.1	*Auriculariaauricula-judae*	XE-987	Y	Y	Y	Y
HQ414243.1	*Auriculariaauricula-judae*	ZHI-5	Y	Y	Y	Y
JF440694.1	*Auriculariaauricula-judae*	HW5D31	Y	Y	Y	Y
JF440695.1	*Auriculariaauricula-judae*	5L0109	Y	Y	Y	Y
JF440696.1	*Auriculariaauricula-judae*	5L0096	Y	Y	Y	Y
JF440735.1	*Auriculariaauricula-judae*	9809	Y	Y	Y	Y
JF440737.1	*Auriculariaauricula-judae*	HE-9	Y	Y	Y	Y
JF440738.1	*Auriculariaauricula-judae*	ME-6	Y	Y	Y	Y
JF440739.1	*Auriculariaauricula-judae*	XE-887	Y	Y	Y	Y
JF440740.1	*Auriculariaauricula-judae*	HE-3	Y	Y	Y	Y
JF440741.1	*Auriculariaauricula-judae*	SHAN-1	Y	Y	Y	Y
JF440742.1	*Auriculariaauricula-judae*	8129	Y	Y	Y	Y
JF440743.1	*Auriculariaauricula-judae*	DA-2	Y	Y	Y	Y
JF440744.1	*Auriculariaauricula-judae*	173	Y	Y	Y	Y
JF440745.1	*Auriculariaauricula-judae*	HME-1	Y	Y	Y	Y
JF440746.1	*Auriculariaauricula-judae*	139	Y	Y	Y	Y
JF440747.1	*Auriculariaauricula-judae*	186	Y	Y	Y	Y
JF440748.1	*Auriculariaauricula-judae*	C21	Y	Y	Y	Y
JF440749.1	*Auriculariaauricula-judae*	CBS-7	Y	Y	Y	Y
JF440750.1	*Auriculariaauricula-judae*	DZ-1	Y	Y	Y	Y
JF440751.1	*Auriculariaauricula-judae*	HEI-29	Y	Y	Y	Y
JF440752.1	*Auriculariaauricula-judae*	SN-A8	Y	Y	Y	Y
JF440753.1	*Auriculariaauricula-judae*	XP-10	Y	Y	Y	Y
JF440754.1	*Auriculariaauricula-judae*	YM-1	Y	Y	Y	Y
JF440755.1	*Auriculariaauricula-judae*	8808	Y	Y	Y	Y
JF440756.1	*Auriculariaauricula-judae*	35431	Y	Y	Y	Y
JF440757.1	*Auriculariaauricula-judae*	DA-1	Y	Y	Y	Y
JF440758.1	*Auriculariaauricula-judae*	DA-3	Y	Y	Y	Y
JF440759.1	*Auriculariaauricula-judae*	JY-1	Y	Y	Y	Y
JF440760.1	*Auriculariaauricula-judae*	ZJ-310	Y	Y	Y	Y
JF440761.1	*Auriculariaauricula-judae*	YE-K3	Y	Y	Y	Y
JF440762.1	*Auriculariaauricula-judae*	HEI-916	Y	Y	Y	Y
JN712676.1	*Auriculariaauricula-judae*	AU110	Y	Y	Y	Y

### ﻿Functional assessment

One of the contigs of the draft genome of *Auriculariaheimuer* (NEKD01000094) was found to contain a ribosomal stretch comprising the expected rRNA genes nuclear small-subunit (nSSU), 5.8S in the ITS region, nuclear large-subunit (nLSU), and 5S but also the RNase P, MRP, and SRP genes, plus a putative U14/SNORD14 ncRNA copy. The result was the same for *Auriculariacornea.* No other copies of these ncRNAs were found in any other part of the genome assembly. *Auriculariasubglabra* strain TFB-10046 SS5 (AFVO01) produced a similar result: we could not identify any of the ncRNAs in the genome assembly. Unfortunately, for this species the rDNA region was not included in the assembly or available elsewhere, but it seems probable that these ubiquitous ncRNAs would be located in the same region as in *A.heimuer*. The same result was obtained for *Auriculariaauricula-judae* strain B14-8 (NCVV01). However, in the genome assembly of *Auriculariapolytricha* strain MG66 (QFEN01), these ncRNAs were found on different contigs and none of these contigs contained any rRNA, implying those are the functional copies should additional ones exist also in the rDNA region. Interestingly, the *A.polytricha* strains AP112 and APFJ do have these ncRNAs in the IGS1 region, but there is no genome assembly available for either AP112 or APFJ.

## ﻿Discussion

Alm Rosenblad et al. (2016) provided the first observation of an ncRNA other than the standard SSU, 5.8S, LSU, and 5S genes – namely the SRP RNA – in the ITS region of fungi. We expand on those findings by highlighting not only the SRP RNA gene but also an additional three ncRNA genes – nuclear RNase P RNA, RNase MRP RNA, and a possible U14 – in the IGS region of several *Auricularia* species. We verified the presence of these IGS ncRNAs through DNA sequencing of conspecific specimens. We furthermore used sequencing to recover the SRP RNA and a putative copy of the U14 gene in the IGS region of three *Inocybe* species. Our findings suggest that the SRP RNA results of Alm Rosenblad et al. (2016) were not isolated occurrences of limited interest to mycology and RNA biology. On the contrary, the SRP RNA gene seems to have been independently and repeatedly incorporated into the ribosomal operon of numerous fungi. This certainly warrants further investigation.

In addition to the SRP RNA, we also found strong evidence for two other ncRNAs in the IGS1 of both *Auricularia* specimens we sequenced – *Auriculariadelicata* (MFLU16-2118) and *Auriculariacornea* (MFLU16-2108) – namely RNase P RNA and RNase MRP RNA. These genes correspond to important components for the maturation of tRNAs and rRNAs, respectively (López et al. 2009). While there are examples of a different type of nuclear RNase P in some organisms, all fungi use the standard RNP-type RNase P (Klemm et al. 2016). For the RNase MRP, there are, as yet, no examples of species lacking this RNP, although the exact composition of its protein subunits is unclear in some groups (Alm Rosenblad et al. 2021). Regarding the U14 snoRNA, which also plays a part in the rRNA maturation process, the prediction score did pass the Rfam threshold, but since a thorough analysis of this ncRNA gene has not yet been made for basidiomycetes, we consider the predictions to be interesting candidates pending further analysis.

The fact that draft genome assemblies of *Auriculariaheimuer* and *A.cornea* contain these RNAs in their ribosomal operon, but not elsewhere in the genome, suggests that these ncRNA genes are functional. Our approach does not enable us to prove that these ncRNAs indeed are functional, but the case for them as functional must be considered strengthened. The RNase P RNA and the RNase MRP RNA genes have been identified in introns of protein coding genes in metazoans such as *Caenorhabditis* and *Drosophila* (López et al. 2009), but there is no example of them from ribosomal operons. However, it has been shown in insects that the RNase P RNA, which is usually transcribed by pol III, is dependent on the recipient gene’s pol II promoter and that splicing is not required for producing a mature RNase P RNA (Manivannan et al. 2015).

Interestingly, whereas our former study found SRP RNAs in the ITS1 region of strictly ectomycorrhizal species, this study reveals the presence of ncRNAs – including the SRP RNA – also in the non-ectomycorrhizal (but instead saprotrophic) basidiomycete genus *Auricularia*. This suggests that fungal nutritional mode may not determine or require the presence of these ncRNAs in the ribosomal operon, something that would be interesting to pursue in light of further data. It should nevertheless be pointed out that all five fungal genera from which ribosomal operon ncRNAs have been reported - *Astraeus*, *Russula*, *Lactarius, Inocybe*, and *Auricularia* – belong to the class Agaricomycetes of the Basidiomycota. The significance of this is unclear, but even a cursory glance at the finer levels of the Basidiomycota phylogeny shows that multiple independent gains/losses are needed to explain the observed ncRNA distribution. The fact that a single fungal class has seen a multitude of these events, whereas no other fungal class seems to have seen even a single one, certainly calls for an explanation.

It seems clear that ncRNAs must be taken into consideration in fungal ribosomal genetics. Four different ncRNAs are now known from the fungal ribosomal operon, and further research should screen genome and RNA operon sequences to determine how widespread ncRNAs are in fungi. Indeed, as databases accumulate a steadily increasing number of fungal ribosomal sequences that go far beyond the ITS region, there is every reason to think that additional ncRNAs will be recovered, presumably from non-Agaricomycetes fungi at that. The ribosomal operon is routinely excluded from many genome assemblies due to assembly difficulties (Hibbett et al. 2016), but our findings stress the importance of including it to enable research efforts like the present one. This study seeks to alert our fellow mycologists and RNA biologists to the presence of these ncRNAs in genetic regions where they up until recently were not expected to be, and we certainly hope that the evolutionary history of these ncRNAs and their presence in the fungal ribosomal operon will prove amenable to scientific explanation within not too long. *Auricularia* mycology primarily relies on genetic markers and genes such as the ITS region, nLSU, and RPB2 (Yuan et al. 2018; Wu et al. 2021), but several studies have explored the *Auricularia* IGS region for mycological usefulness (e.g., Li et al. 2011 and Li et al. 2019). The present study suggests that caution is warranted when aligning the IGS region of *Auricularia* and possibly also other fungi due to the potential presence of these ncRNAs. Uncritical alignments may violate homology assumptions and may give rise to noisy multiple sequence alignments and skewed phylogenetic signals.

## ﻿Conclusions

This study reports on the detection of three non-coding RNA genes hitherto not known from the fungal ribosomal region: nuclear RNase P RNA, RNase MRP RNA, and a possible snoRNA U14 in a total of five species of *Auricularia* and *Inocybe*. This expands on the recent finding of another non-coding RNA gene – the Signal Recognition Particle (SRP) RNA – in the internal transcribed spacer (ITS) region of three ectomycorrhizal genera of basidiomycetes. There are indications that these are functional genes rather than pseudogenes. The occurrence of these non-coding RNAs and their distribution in the fungal tree of life calls for further research attention but also caution in, e.g., multiple sequence alignment-based phylogenetic inference efforts involving the ribosomal regions of these fungi.

## Supplementary materials

Supplementary material 1 Details of the sequence processing steps. Data type pdf file Explanation note Details of the sequence processing steps. https://binary.pensoft.net/file/708913This dataset is made available under the Open Database License (http://opendatacommons.org/licenses/odbl/1.0/). The Open Database License (ODbL) is a license agreement intended to allow users to freely share, modify, and use this Dataset while maintaining this same freedom for others, provided that the original source and author(s) are credited.Magnus Alm Rosenblad, Ellen Larsson, Arttapon Walker, Naritsada Thongklang, Christian Wurzbacher, R. Henrik Nilsson

Supplementary material 2 List of absolute positions of the rRNA and ncRNA genes in the six sequences released with this study Data type pdf file Explanation note List of absolute positions of the rRNA and ncRNA genes in the six sequences released with this study. The positions listed are from Infernal cmscan searches and the names are those used by Rfam. For *Auricularia*, the special model for basidiomycete SRP RNA from Dumesic et al. (2015) was used. Positions for the WGS contig of *Auriculariaheimuer* mentioned in the text has been added below the sequences from this study. Note that the contig has been reverse-complemented before annotation to aid comparison. The contig contains four rRNA operons. https://binary.pensoft.net/file/708914This dataset is made available under the Open Database License (http://opendatacommons.org/licenses/odbl/1.0/). The Open Database License (ODbL) is a license agreement intended to allow users to freely share, modify, and use this Dataset while maintaining this same freedom for others, provided that the original source and author(s) are credited.Magnus Alm Rosenblad, Ellen Larsson, Arttapon Walker, Naritsada Thongklang, Christian Wurzbacher, R. Henrik Nilsson
