# Supplementary material for: ﻿Evidence for further non-coding RNA genes in the fungal rDNA region
Source: MycoKeys. 2022 Jun 30;90:203–13. doi: 10.3897/mycokeys.90.84866 (PMC9849065; doi:10.3897/mycokeys.90.84866)
Supplement: Supplementary material 1 — Details of the sequence processing steps. [file mycokeys-90-203-s001.pdf]

Supplementary Item 1 – Details of the sequence processing steps.

This document is a brief summary of select parts of the Materials and Methods section of Wurzbacher et al. (2019), to which the reader should turn for the full details.

Primer design

The primer pair NS1short and RCA95m was used to amplify the greater part of the ribosomal operon of the ribosomal tandem repeat. The reverse complementary version of each primer was used to amplify the missing parts of the ribosomal region (the 3′ end of the LSU, IGS, and the ETS region). As a third primer pair, we used the forward primer Fun-rOP-F that amplifies in the 3′ direction of the LSU, while the reverse primer Fun-rOP-R amplifies in the 5′ direction. The last four nucleotides of both Fun-rOP primers are pairing, and these four nucleotides resemble the position overlap in the template (CTGA) at the exact *Escherichia coli* reference position 1770–1773 of the LSU (SILVA LSU reference position). This allows an end-to-end assembly of the full ribosomal region (LSU-IGS-ETS-SSU-ITS1-5.8S-ITS2-LSU) extracted from the ribosomal tandem repeat. All primer pairs were barcoded following the dual indexing strategy of Illumina sequencing, that is, we introduced the forward barcode series S500 to the 5′ end of each forward primer and the N700 barcode series to the 5′ end of the reverse primer. This allows simultaneous sequencing of more than 100 samples. After each barcode, one or two extra nucleotides were added as a precaution against nuclease activity. In between barcode and primer nucleotides we added a two-nucleotide wide spacer that has a mandatory mismatch to the fungal kingdom at these two positions.

Long-range PCRs

We used the PrimeStar GLX polymerase (Takara) for all primer systems. The PCR was performed in 40 µl reactions with 1.5 µl enzyme, 12 pmol of each barcoded primer (a unique combination for each sample), 1 mM dNTPs, and 1 µl of template (with a concentration of approximately 1–40 ng/µl). For all primer pairs, we ran an initial denaturation of 1 min at 98°C, then 36 cycles at 98°C for 10 s, 55°C for 15 s, and 68°C for 2.5 min. We increased the elongation step of the TR PCR from 2.5 to 4 min. An initial PCR with native (non-barcoded) NS1short/RCA95m primers and 3% BSA (molecular grade, Carl-Roth) as additive was run with the following program: 5 min at 95°C, then 35 cycles at 95°C for 30 s, 55°C for 30 s, and 68°C for 4 min. The PCR product was then used as template in a second PCR with 10 cycles but otherwise identical conditions, exchanging the native primers with barcoded primers.

Library preparation and sequencing

The PCR products were purified with either 0.8 (v/v) of AMPure beads (Beckmann) or PCR purification plates (Qiagen) following the respective manufacturer's recommendations. After that, the purified PCR products were quantified using Nanodrop 2000 (Thermo Scientific) and pooled in an approximately equimolar way. This final pool was purified with AMPure beads using 0.4 (v/v) of beads and eluted in 50–100 µl molecular grade water. The concentration of the amplicon pool was quantified with a Qubit instrument (Invitrogen).

Approximately 2–4 µg was sent for sequencing with PacBio RSII (Pacific Biosciences) at the Swedish SciLife Lab in Uppsala, Sweden. Another batch of 800 ng was used for Oxford Nanopore library preparation following the manufacturer's protocol for sequencing (LSK-208; LSK-308; Oxford Nanopore Technologies). Sequencing was done locally on a MinION instrument (Oxford Nanopore Technologies) operated with FLO-107 flowcells (with the R9.5

chemistry). We stopped the sequencing as soon as we reached 2,000 sequences, which took 2–8 h depending on the pool size and amplicon length.

Data processing

2D base calling was done with Albacore (v2.4; Oxford Nanopore Technologies). Unpaired reads were discarded. For the PacBio data, we only worked with the "reads of insert" (ROS) data from the circular consensus sequencing mode. After these initial steps, all sequences from both sequencing platforms were processed in the same way.

An initial quality-filtering step (USEARCH v8.1; Edgar 2010) was performed at an error rate of 0.02 for PacBio sequences while Nanopore reads were already filtered at the error rate 0.08. Afterwards, all sequences were filtered by length using Biopython (v1.65; Cock et al. 2009) to exclude overly short and long sequences as detected in the histograms. All quality-filtered and trimmed sequences were demultiplexed as FASTA files into individual samples according to their combined barcodes (Flexbar, v2.5; Dodt et al. 2012). Barcodes and adapters were removed in this step. All sequences from each individual sample were aligned in MAFFT (v7.397; Katoh and Standley 2013). The aligned sequences were clustered in mothur (v1.39; Schloss et al. 2009) using the Opticlust algorithm, and the consensus sequences for each operational taxonomic unit (OTU; Blaxter et al. 2005) were inferred using a custom-made Perl script (Consension; https://microbiology.se/software/consension/). The optimal OTU clustering threshold for Nanopore data was determined to be 0.07 for shorter amplicons (rDNA and IGS PCR) and 0.08 for the long TR PCR (see Wurzbacher et al. (2019) for more details). To counter spurious OTUs, we determined a dynamic OTU size cut-off that was provided to Consension. It was calculated as:

K = (number of sample reads * error rate) / (length correction)

This integer is defined as amplicon length [kb] divided by 5 kb of the expected amplicon length. Kmin (the minimum number of sequences a OTU can hold) was set to 3 for PacBio and 5 for Nanopore sequences. The consensus sequences were compared by inspecting the alignment visually in SeaView (v.4.7; Gouy et al. 2009) and by computing sequence similarities with local BLAST searches (nucleotide BLAST+, v2.2.28). In the few cases where we obtained more than 1 OTU after consensus generation, we only used the most abundant OTU for subsequent similarity comparisons.

References

Blaxter M, Mann J, Chapman T, Thomas F, Whitton C, Floyd R, Abebe E (2005) Defining operational taxonomic units using DNA barcode data. Philosophical Transactions of the Royal Society of London series B Biological Sciences 360(1462): 1935-1943. doi: 10.1098/rstb.2005.1725.

Cock PJ, Antao T, Chang JT, Chapman BA, Cox CJ, Dalke A, Friedberg I, Hamelryck T, Kauff F, Wilczynski B, de Hoon MJL (2009) Biopython: Freely available Python tools for computational molecular biology and bioinformatics. Bioinformatics 25(11): 1422–1423. doi: 10.1093/bioinformatics/btp163

Dodt M, Roehr JT, Ahmed R, Dieterich C (2012) FLEXBAR—flexible barcode and adapter processing for next-generation sequencing platforms. Biology 1(3): 895–905. doi: 10.3390/biology1030895

Edgar RC (2010) Search and clustering orders of magnitude faster than BLAST. Bioinformatics 26(1): 2460–2461. doi: 10.1093/bioinformatics/btq461

Gouy M, Guindon S, Gascuel O (2009). SeaView version 4: A multiplatform graphical user interface for sequence alignment and phylogenetic tree building. Molecular Biology and Evolution 27(2): 221–224. doi: 10.1093/molbev/msp259

Katoh K, Standley DM (2013) MAFFT multiple sequence alignment software version 7: Improvements in performance and usability. Molecular Biology and Evolution 30(4): 772–780. doi: 10.1093/molbev/mst010

Schloss PD, Westcott SL, Ryabin T, Hall JR, Hartmann M, Hollister EB, Lesniewski RA, Oakley BB, Parks DH, Robinson CJ, Sahl JW, Stres B, Thallinger GG, van Horn DJ, Weber CF (2009) Introducing mothur: Open-source, platform-independent, community-supported software for describing and comparing microbial communities. Applied and Environmental Microbiology 75(23): 7537–7541. doi: 10.1128/AEM.01541-09

Wurzbacher C, Larsson E, Bengtsson-Palme J, van den Wyngaert S, Svantesson S, Kristiansson E, Kagami M, Nilsson RH (2019) Introducing ribosomal tandem repeat barcoding for fungi. Molecular Ecology Resources 19(1): 118-127. doi: 10.1111/1755-0998.12944
