# Supplementary material for: ﻿Evidence for further non-coding RNA genes in the fungal rDNA region
Source: MycoKeys. 2022 Jun 30;90:203–13. doi: 10.3897/mycokeys.90.84866 (PMC9849065; doi:10.3897/mycokeys.90.84866)
Supplement: Supplementary material 2 — List of absolute positions of the rRNA and ncRNA genes in the six sequences released with this study [file mycokeys-90-203-s002.pdf]

Supplementary item 2 - List of absolute positions of the rRNA and ncRNA genes in the six sequences released with this study. The positions listed are from Infernal cmscan searches and the names are those used by Rfam. For *Auricularia* the special model for basidiomycete SRP RNA from Dumesic et al. (2015) was used. The WGS contig for *Auricularia heimuer* mentioned in the text has been added below the sequences from this study. Note that the contig has been reverse-complemented before annotation to aid in comparisons. The contig contains four rRNA operons.

OM964554 – *Inocybe leiocephala* EL85-16(nanopore)

LSU_rRNA_eukarya	<1	2029	+
RNaseP_nuc	2294	2628	+
SNORD14	2684	2916	+
5S_rRNA	3068	3187	+
SSU_rRNA_eukarya	6983	9165	+
5_8S_rRNA	9434	9587	+
LSU_rRNA_eukarya	9831	11976>	+

OM964555 - *Inocybe cincinnata* EL113-16(pacbio)

LSU_rRNA_eukarya	<1	2031
RNaseP_nuc	2102	2393
SNORD14	2466	2693
5S_rRNA	2799	2917
SSU_rRNA_eukarya	5231	7410
5_8S_rRNA	7667	7820
LSU_rRNA_eukarya	8031	10175>

OM964556 - *Inocybe flocculosa* EL168-16 (pacbio)

LSU_rRNA_eukarya	<1	1647	+
RNaseP_nuc	1716	1995	+
SNORD14	2282	2502	+
5S_rRNA	2749	2867	+
SSU_rRNA_eukarya	6026	8211	+
5_8S_rRNA	8451	8604	+
LSU_rRNA_eukarya	8808	10948>	+

OM964557 - *Inocybe phaeocystidiosa* EL23-16 (pacbio)

LSU_rRNA_eukarya	<1	1257
5S_rRNA	1930	2048
SSU_rRNA_eukarya	5592	7397
5_8S_rRNA	7583	7736
LSU_rRNA_eukarya	7936	10091>

OM964558 - *Auricularia cornea* MFLU16-2108 (nanopore)

LSU_rRNA_eukarya	<1	1254	+	
RNaseP_nuc	1637	1946	+	
RNase_MRP	2253	2550	+	
Fungi_SRP	2891	2621	-	[NOTE positions Minus strand, basidio cm from Dumesic et al. 2015]
SNORD14	2967	3165	+	
5S_rRNA	3522	3640	+	
SSU_rRNA_eukarya	6339	6370>	+	

OM964559 - *Auricularia delicata* MFLU16-2118(nanopore)

LSU_rRNA_eukarya	<1	1254	+	
RNaseP_nuc	1649	1958	+	
RNase_MRP	2271	2555	+	
Fungi_SRP	2885	2612	-	[NOTE positions Minus strand , basidio cm from Dumesic et al. 2015]
SNORD14	2958	3154	+	
5S_rRNA	3532	3650	+	
SSU_rRNA_eukarya	5841	5872>	+	

NEKD01000094.1-REVERSED - *Auricularia heimuer* Dai 13782 [Length: 40944 bp]

LSU_rRNA_eukarya	<1	1496	+	
RNaseP_nuc	1851	2161	+	
RNase_MRP	2420	2668	+	
Fungi_SRP	2960	2691	-	[cm by Dumesic et al.]
SNORD14	3028	3229	+	

5S_rRNA	3595	3713	+

SSU_rRNA_eukarya	7056	8860	+
5_8S_rRNA	9018	9171	+
LSU_rRNA_eukarya	9372	12762	+
RNaseP_nuc	13117	13427	+
RNase_MRP	13686	13934	+
Fungi_SRP	14226	13957	- [cm by Dumesic et al.]
SNORD14	14294	14495	+
5S_rRNA	14861	14979	+

SSU_rRNA_eukarya	18321	20125	+
5_8S_rRNA	20283	20436	+
LSU_rRNA_eukarya	20637	24027	+
RNaseP_nuc	24382	24692	+
RNase_MRP	24951	25199	+
Fungi_SRP	25491	25222	-
SNORD14	25559	25760	+
5S_rRNA	26126	26244	+

SSU_rRNA_eukarya	29586	31390	+
5_8S_rRNA	31548	31701	+
LSU_rRNA_eukarya	31902	35292	+
RNaseP_nuc	35647	35957	+
RNase_MRP	36216	36464	+
Fungi_SRP	36756	36487	- [cm by Dumesic et al.]
SNORD14	36824	37025	+
5S_rRNA	37391	37509	+

SSU_rRNA_eukarya   40849   40944>   +

//

References

Dumesic PA, Rosenblad MA, Samuelsson T, Nguyen T, Moresco JJ, Yates JR, Madhani HD (2015) Noncanoncial signal recognition particle RNAs in a major eukaryotic phylum revealed by purification of SRP from the human pathogen *Cryptococcus neoformans*. Nucleic Acids Research 43(18): 9017-9027. doi: 10.1093/nar/gkv819
